# Nomogram model predicts the risk of visual impairment in diabetic retinopathy: a retrospective study

**DOI:** 10.1186/s12886-022-02710-6

**Published:** 2022-12-08

**Authors:** Yuancheng Zhao, Rentao Yu, Chao Sun, Wei Fan, Huan Zou, Xiaofan Chen, Yanming Huang, Rongdi Yuan

**Affiliations:** 1 Department of Ophthalmology, the Second Affiliated Hospital of Army Medical University, 183#, Xinqiaozheng St., Shapingba District, Chongqing, 400037 People’s Republic of China; 2 Department of Dermatology, the First Affiliated Hospital of Chongqing Medical University, 1#, Youyi Road, Yuanjiagang, Yuzhong District, Chongqing, China

**Keywords:** Nomogram, Visual impairment, Decision curve analysis, Clinical impact curve, Optical coherence tomography angiography, Diabetic retinopathy

## Abstract

**Background:**

To develop a model for predicting the risk of visual impairment in diabetic retinopathy (DR) by a nomogram.

**Methods:**

Patients with DR who underwent both optical coherence tomography angiography (OCTA) and fundus fluorescein angiography (FFA) were retrospectively enrolled. FFA was conducted for DR staging, swept-source optical coherence tomography (SS-OCT) of the macula and 3*3-mm blood flow imaging by OCTA to observe retinal structure and blood flow parameters. We defined a logarithm of the minimum angle of resolution visual acuity (LogMAR VA) ≥0.5 as visual impairment, and the characteristics correlated with VA were screened using binary logistic regression. The selected factors were then entered into a multivariate binary stepwise regression, and a nomogram was developed to predict visual impairment risk. Finally, the model was validated using the area under the receiver operating characteristic (ROC) curve (AUC), calibration plots, decision curve analysis (DCA), and clinical impact curve (CIC).

**Results:**

A total of 29 parameters were included in the analysis, and 13 characteristics were used to develop a nomogram model. Finally, diabetic macular ischaemia (DMI) grading, disorganization of the retinal inner layers (DRIL), outer layer disruption, and the vessel density of choriocapillaris layer inferior (SubVD) were found to be statistically significant (*P* < 0.05). The model was found to have good accuracy based on the ROC (AUC = 0.931) and calibration curves (C-index = 0.930). The DCA showed that risk threshold probabilities in the (3–91%) interval models can be used to guide clinical practice, and the proportion of people at risk at each threshold probability is illustrated by the CIC.

**Conclusion:**

The nomogram model for predicting visual impairment in DR patients demonstrated good accuracy and utility, and it can be used to guide clinical practice.

**Trial registration:**

Chinese Clinical Trial Registry, ChiCTR2200059835. Registered 12 May 2022,

https://www.chictr.org.cn/edit.aspx?pid=169290&htm=4

**Supplementary Information:**

The online version contains supplementary material available at 10.1186/s12886-022-02710-6.

## Background

Diabetes is one of the fastest-growing chronic diseases in the world [1], and diabetic retinopathy (DR) is one of the major causes of vision loss in diabetic patients [2]. Studies have been performed on early risk screening for type 1 diabetes [3], type 2 diabetes [4] and DR [5], and the initial results will be progressively applied to clinical practice [6]. Currently, fundus fluorescein angiography (FFA) is the gold standard for the diagnostic grading of DR [7], and swept-source optical coherence tomography (SS-OCT) can construct clear two-dimensional cross-sectional images of the retina [8]. The novel recently developed optical coherence tomography angiography (OCTA) procedure is very sensitive to retinal microvascular changes, and it can yield clear images among some patients with poorly defined refractive media (cataract, vitreous opacities) [9].

It has been shown that DR staging [2] and retinal structural abnormalities (disorganization of the retinal inner layers (DRIL) [10], diabetic macular ischaemia (DMI) [11], diabetic macular oedema (DME) [12], outer layer disruption [13], structural changes in the foveal avascular zone (FAZ) [14], etc.) are correlated with visual impairment in DR patients, but no studies have revealed the magnitude of the influence of various correlated factors in the formation of visual impairment in DR patients. Furthermore, no studies have integrated these risk factors to construct an efficient, accurate, simple and intuitive model for predicting the risk of visual impairment. A nomogram would be useful to achieve this goal. Based on the reported risk factors for visual acuity (VA) in DR patients and some new retinal structure indicators, this study screened out risk factors that were strongly correlated with VA through regression analysis and creatively integrated and quantified the impact of the risk factors for visual impairment in DR patients using a nomogram [15]. By scoring each factor, a convenient and practical risk prediction model for visual impairment was constructed to guide clinical decision-making.

## Methods

### Subjects

This research was implemented in accordance with the requirements of the Declaration of Helsinki, and the protocol was approved by the Ethics Committee of the Second Affiliated Hospital of Army Medical University. This study was registered in the Clinical Trials Registry (ChiCTR2200059835). A total of 252 eyes in 133 patients with DR who attended the ophthalmology department of the Second Affiliated Hospital of the Army Medical University were examined from August 2020 to January 2022. The inclusion criteria were as follows: 1. patients of either sex, ages ≥18 years; 2. patients diagnosed with DR according to the International Clinical Classification Criteria for Diabetic Retinopathy (2002) [16]; and 3. clear images were obtained for patients who had undergone OCTA and FFA within 1 week. The exclusion criteria were as follows: 1. patients with other fundus retinopathies in addition to DR (age-related macular degeneration, polypoid chorioretinopathy, optic nerve atrophy, etc.); 2. patients with refractive media clouding (cataracts affecting vision, vitreous haemorrhage) for which clear images cannot be obtained; and 3. patients with other ophthalmic diseases affecting vision. The exclusion process and reasons for all exclusions are shown in Online Additional file 1.

### General examination

The patient’s best-corrected visual acuity (BCVA) was assessed using the International Standard Visual Acuity Scale and converted to logarithm of the minimum angle of resolution (LogMAR) VA. Slit lamp, intraocular pressure (IOP) and fundus photograph were routinely examined, and the patient’s duration of diabetes at this visit, comorbidities, and examination test results were recorded.

### OCTA and OCT image acquisition and processing

The OCTA instrument (DRI OCT Triton; Topcon Inc., Tokyo, Japan) we utilize used the latest technology. The device type was SS-OCT, which was based on the optical coherence tomography angiography ratio analysis (OCTARA) algorithm, the wavelength was 1050 nm, the acquisition speed was 100 kHz, and the axial and lateral resolution of the tissue was 7 μm and 20 μm, respectively. The capillary plexus was automatically segmented as follows (IMAGEnet6): the superficial capillary plexus (SCP) was located 3 mm below the internal limiting membrane (ILM) to 15 mm below the junction of the inner plexiform layer (IPL) and inner nuclear layer (INL); the deep capillary plexus (DCP) was located 15 mm below the IPL/INL to 70 mm below the INL, and the choriocapillaris layer was located Bruch Membrane (BM) to 20 mm below the BM [17]. The following images were excluded: (1) quality scores < 45; (2) blurred images (inability to distinguish capillaries from background signal); (3) artefacts (white lines, vessel displacement) due to movement and blinking; and (4) images that did not distinguish between DCP and SCP. SS-OCT of the macula was performed on the patient and analysed for DRIL, DME occurrence, and outer layer disruption of the retina (Online Additional file 2). A 3*3-mm (each b-scan includes 320 A-scans for a total of 320 b-scans) flow imaging was performed from SCP, which was used to evaluate DMI grading in artificial means (Online Additional file 3) [18], and foveal avascular zone (FAZ) (d = 1 mm) and paracentral fovea (d = 0.75 mm) vessel density (VD) origin in DCP and SCP were analysed using IMAGEnet6 software. FAZ area, perimeter [19], circularity [20] (Online Additional file 4), and nonperfusion area (NPA) within 3 mm*3 mm [21] of DCP and SCP were analysed using ImageJ software (Online Additional file 5) (Table 1).

**Table 1:** Definition of OCT/OCTA Metrics

Parameters	Unit	Definition
**Foveal Avascular Zone**		
Area	mm^2^	Area within manually traced FAZ.
Perimeter	mm	Perimeter of manually traced FAZ
Circularity		Irregularity of the FAZ perimeter when compared to the perimeter of a perfect circle of equal area. Mathematically, it is defined by the formula: 4π*[Area]/[Perimeter]^2^，with a value of 1.0 indicating a perfect circle.
**Disorganization of the Retinal Inner Layers**	Grade	Unidentifiable boundaries of the ganglion cell layer (GCL)—inner plexiform layer (IPL) complex, inner nuclear layer (INL), and outer plexiform layer (OPL) within the central 3000 μm region. Grade0 = none；Grade1 = single Side of the FAZ；Grade2 = both Sides of the FAZ.
**Outer Layer Disruption**	Yes	Outer layer disruption was defined as horizontal length of disruptions involving the external limiting membrane (ELM) 、Ellipsoid zone (EZ) and Retinal pigment epithelium (RPE).
**Diabetic Macular Ischemia**	Grade	Grade 1 = contour not smooth, round, or oval, visible irregularities, but not necessarily abnormal. Grade 2 = obvious damage, < 180°; Grade 3 = obvious damage, > 180°, partial remnants; Grade 4 = complete damage to the contour.
**Diabetic Macular Edema**	Yes	Defined as a CRT > 300 μm.The presence of intraretinal cysts was defined as the presence of localized hypo reflective areas, only the presence or absence of cystoid macular edema was observed, and no degree of grading was performed.
**Vessel Density**	%	Ratio of white pixels (vessels/flow) to total pixels in an image.
**Non-perfusion Area**	mm^2^	Using the binarization and inversion functions of ImageJ software, the gap between adjacent blood vessels was defined as the non-perfusion area, and the total area of the non-perfusion area within the range of 3*3 mm was directly measured.

### FFA image

FFA was performed on the patient using a Heidelberg (SPECTRALIS HRA), arm retinal time (ART) was recorded and DR staging was performed on the affected eye [22].

### Reproducibility

Manual outlining of the images was performed using ImageJ (version 1.51a), and the grade was assessed by two blinded specialists (Chen and Zou). The specialists showed good agreement for manual measurement indicators (Online Additional file 6), and disagreements were resolved via discussion.

### Statistical analysis

SPSS 26.0 (IBM Corporation, New York, USA) and the ‘rms’ and ‘rmda’ packages of RStudio (version 4.1.3; https://www.R-project.org) software were used for statistical analysis. The normality of the measurement data was determined using the Kolmogorov–Smirnov test, and those that did not satisfy the normal distribution were expressed as medians (quartiles). The enumeration data were presented as frequencies (percentages). LogMAR VA was transformed into binary outcomes with or without impairment according to the cut-off value criterion (Standard: Blindness and Visual Impairment Criteria (International Classification of Diseases, WHO, 2009) defining VA ≥ 0.5 as having impaired vision and the opposite is not) [23], and each parameter was subjected to univariate logistic regression analysis and validated using the area under the receiver operating characteristic (ROC) curve (AUC). The parameters that were strongly correlated with VA were subsequently entered into a multivariate logistic regression (LR) in a stepwise manner to construct the nomogram. The model accuracy and fit were assessed using ROC and calibration curves, and decision curve analysis (DCA) [24] was conducted to assess the rate of the benefit of the model to patients. Clinical impact curve (CIC) was used to stratify risk proportions for each threshold probability in the model [25]. *P* < 0.05 was considered to indicate a statistically significant difference.

## Results

### Demographic data

Of the 133 patients, the median age was 56 years (50–61 years), the median duration of diabetes was 10 years (7–16 years), the median HbA1c value was 8.6% (7.3–10.5%), hypertension was observed in a total of 76 patients (57.14%), and diabetic nephropathy was observed in a total of 77 patients (59.69%). Table 2 showed the underlying clinical characteristics of the included patients. There were 252 eyes with nonproliferative diabetic retinopathy (NPDR) lesions, 49 (19.44%) eyes with mild DR, 89 (35.32%) eyes with moderate DR, 53 (21.03%) eyes with severe DR, and 61 (24.21%) eyes with proliferative diabetic retinopathy PDR.

**Table 2:** Demographics characteristics of patients (*N* = 133)

Parameters	Number	Mid (IQR) or %
Age, years	133	56(50–61)
Gender, men	85	63.91%
Duration of diabetes, years	129	10(7–16)
ART, seconds	133	15(14–19)
Smoking, yes	46	35.66%
Drinking, yes	26	20.16%
**Comorbidities,** yes		
Hypertension	76	57.14%
DN	77	59.69%
Hyperlipidemia	39	30.23%
Hypercholesterolemia	10	7.75%
Hyperuricemia	19	15.20%
Cardiovascular disease	28	21.71%
Anemia	17	13.18%
**Laboratory parameters**		
UCV, Abnormal	82	72.57%
eGFR, ml/min/L	125	88(56–102)
TG, mmol/L	123	1.48(1.02–2.29)
TCH, mmol/L	123	4.47(3.66–5.37)
HDL-C, mmol/L	123	1.04(0.885–1.27)
LDL-C, mmol/L	123	2.22(1.63–2.98)
HbA1c, %	126	8.6(7.3–10.5)

*Abbreviations*: *ART* arm retinal time, *DN* diabetic nephropathy, *UCV* ultrasound of cervical vascular, *TG* triglycerides, *TCH* total cholesterol, *HDL-C* high density lipoprotein cholesterol, *LDL-C* low-density lipoprotein cholesterol, *eGFR* estimated glomerular filtration rate

### Development of the nomogram models

A total of 44 eyes with impairment and 206 eyes without impairment were examined, indicating an impairment rate of 17.5%. First, each risk factor was analysed by univariate binary regression (Table 3), and risk factors with statistical significance (*P* < 0.05) and AUC > 0.650 in both binary regression and ROC curve analysis were screened. The following factors were included in the regression model: DR, DMI, DME, DRIL, outer layer disruption, NPA (SCP, SCP + DCP), VD (superior of SCP, centre and superior of DCP, inferior of choriocapillaris), FAZ (area and perimeter of SCP), and systemic factors such as sex, age, HbA1c, EGFR, TG, TCH, duration of diabetes, and the presence of hypertension and diabetic nephropathy. The model was statistically significant (*P* < 0.001) (Table 4).

**Table 3:** LogMAR VA impairment correlation with various parameters (*N* = 252)

Parameters	OR (95%CI)	*P*	AUC (95%CI)	*P*
DR Staging	0.13 (0.05–0.34)	0.000	0.776(0.711–0.840)	0.000
**OCT Metrics**				
DME	7.37(1.59–4.09)	0.011	0.770(0.683–0.857)	0.000
DRIL	19.08(8.59–42.36)	0.000	0.801(0.718–0.885)	0.000
Outer Layer Disruption	16.50(6.92–39.36)	0.000	0.703(0.605–0.802)	0.000
**OCTA Metrics**				
DMI	24.90(3.29–188.42)	0.002	0.825(0.764–0.887)	0.000
**Vessel Density(%)**				
**SCP** Center	0.96(0.89–1.04)	0.310	0.560(0.460–0.660)	0.207
Superior	0.89(0.82–0.96)	0.004	0.660(0.560–0.760)	0.001
Inferior	0.93(0.86–1.00)	0.052	0.597(0.502–0.692)	0.044
Nasal	0.98(0.90–1.06)	0.684	0.527(0.416–0.637)	0.578
Temporal	0.93(0.85–1.01)	0.078	0.569(0.466–0.671)	0.153
**DCP** Center	1.15(1.09–1.22)	0.000	0.702(0.599–0.805)	0.000
Superior	0.88(0.82–0.96)	0.002	0.661(0.561–0.762)	0.001
Inferior	0.93(0.87–1.00)	0.036	0.629(0.529–0.729)	0.007
Nasal	0.93(0.86–1.01)	0.083	0.566(0.464–0.667)	0.171
Temporal	0.96(0.90–1.02)	0.175	0.516(0.413–0.618)	0.740
**Choriocapillaris layer** Center	0.91(0.86–0.97)	0.002	0.630(0.536–0.723)	0.007
Superior	0.85(0.77–0.94)	0.002	0.638(0.548–0.728)	0.004
Inferior	0.80(0.73–0.88)	0.000	0.699(0.612–0.787)	0.000
Nasal	0.89(0.80–0.98)	0.018	0.576(0.471–0.680)	0.115
Temporal	0.87(0.80–0.94)	0.001	0.650(0.550–0.749)	0.002
**Foveal Avascular Zone**				
Area SCP(mm^2^)	20.13(5.18–78.28)	0.000	0.690(0.600–0.781)	0.000
DCP	3.78(1.74–8.22)	0.001	0.639(0.536–0.742)	0.004
Perimeter SCP(mm)	1.83(1.37–2.44)	0.000	0.682(0.592–0.772)	0.000
DCP	1.00(1.00–1.00)	0.438	0.688(0.593–0.783)	0.000
Circularity SCP	0.08(0.01–0.68)	0.021	0.581(0.487–0.676)	0.090
DCP	0.01(0.00–0.06)	0.000	0.687(0.604–0.771)	0.000
NPA in 3*3 mm(mm^**2**^**)**				
SCP	2.60(1.35–5.01)	0.004	0.690(0.611–0.768)	0.000
DCP	1.86(1.00–3.47)	0.050	0.635(0.534–0.736)	0.005
SCP + DCP	1.52(1.10–2.11)	0.012	0.664(0.572–0.755)	0.001

***Abbreviations*:** *DR* diabetic retinopathy, *DMI* diabetic macular ischemia, *DME* diabetic macular edema, *DRIL* disorder retinal inner layer, *Outer layer disruption* retinal pigment epithelium or ellipsoid zone rupture, *SCP* superficial capillary plexus, *DCP* deep capillary plexus, *NPA* no perfusion area

**Table 4:** Statistical analysis results of predictive model

Parameters	Unit	β	SE	Wald	***P***	OR (95%CI)
DMI	Grading	3.067	1.143	7.197	0.007	21.49(2.29–202.00)
SubVD	%	−0.207	0.072	8.399	0.004	0.81(0.71–0.94)
DRIL	Grading	2.095	0.547	14.644	0.000	8.12(2.78–23.75)
Outer Layer Disruption	Yes	2.121	0.669	5.431	0.020	4.01(1.25–12.90)

*Abbreviations*: *DMI* diabetic macular ischemia, *DRIL* disorder retinal inner layer, *SubVD* vessel density of choriocapillaris layer Inferior, *Outer layer disruption* retinal pigment epithelium or ellipsoid zone rupture

### Validation of nomogram models

**Fig. 1:**
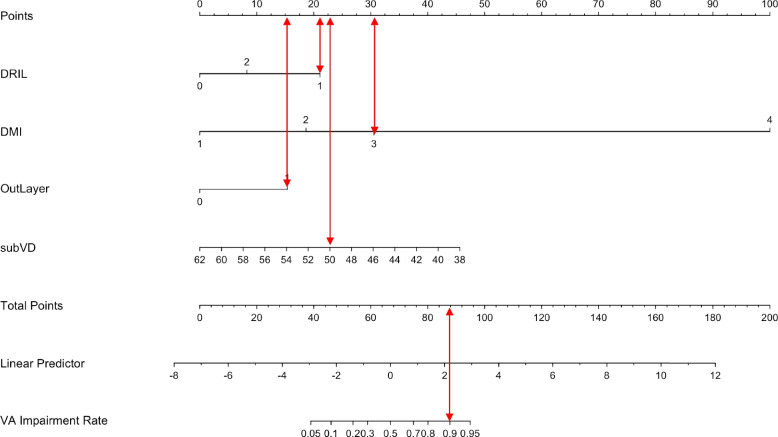
Risk nomogram Model. The VA impairment risk nomogram was developed with the predictors DRIL, DMI, outer layer disruption, and SubVD. DMI = diabetic macular ischaemia; DRIL = disorder retinal inner layer; SubVD = vessel density of choriocapillaris layer inferior

**Fig. 2:**
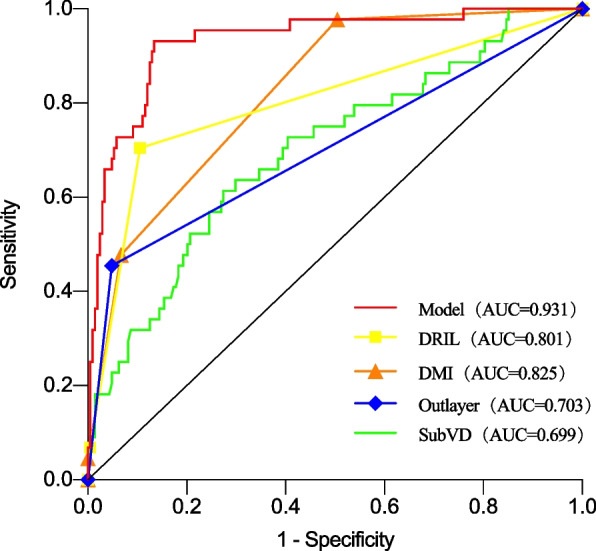
Receiver operating characteristic curve (ROC) curve validation of parameters affecting visual acuity. The y-axis indicates the true-positive rate of the risk prediction. The x-axis indicates the (1-true negative) rate of the risk prediction. The constructed model has higher accuracy than the individual risk factors. DMI = diabetic macular ischaemia; DRIL = disorder retinal inner layer; SubVD = vessel density of choriocapillaris layer inferior; Outer layer disruption = retinal pigment epithelium or ellipsoid zone rupture

**Fig. 3:**
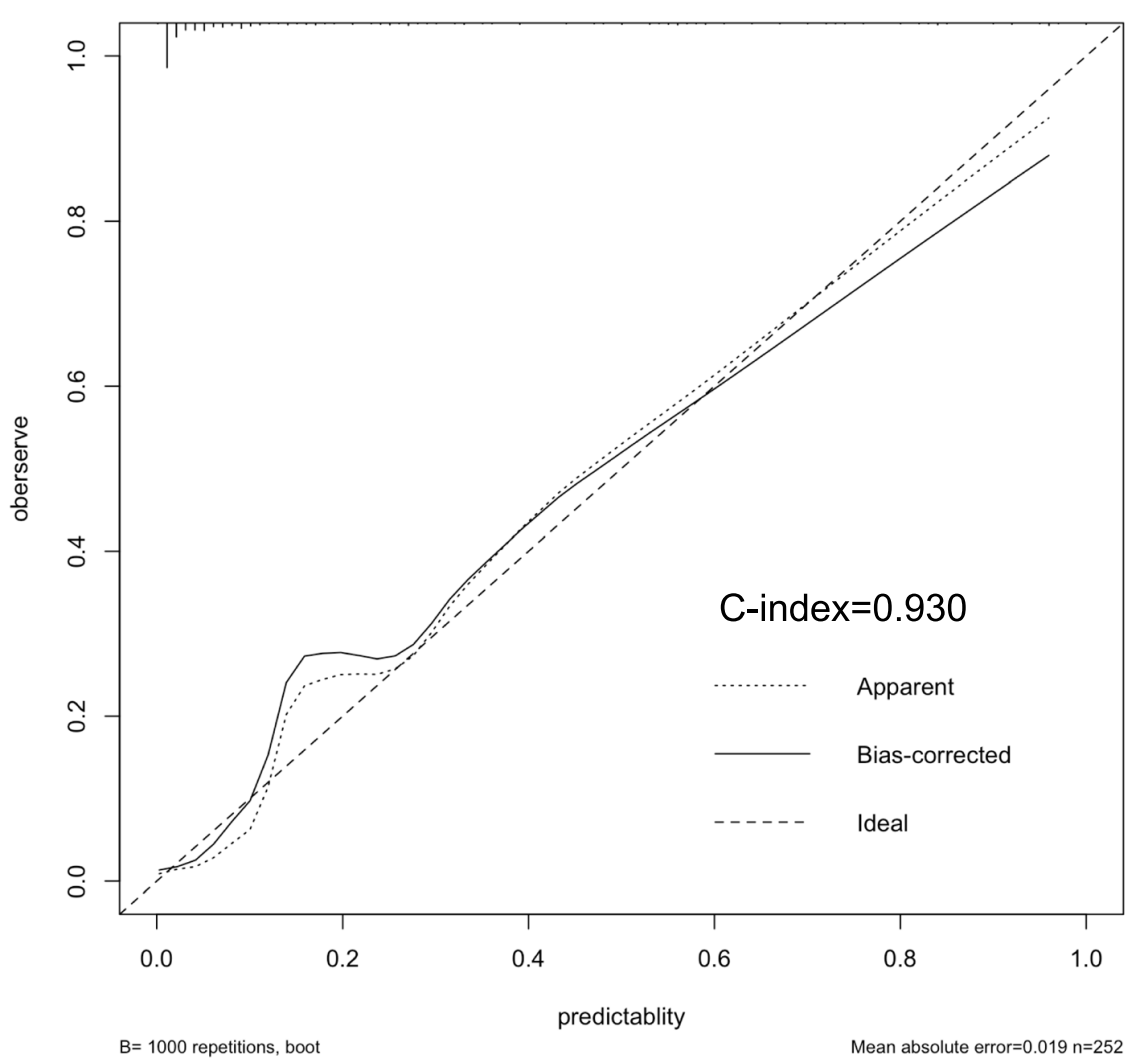
Calibration curves of the VA impairment risk nomogram prediction. The y-axis indicates the actual diagnosed VA impairment. The x-axis indicates the predicted risk of VA impairment. The diagonal dotted line indicates a perfect prediction by an ideal model. The solid line represents the performance of the model, which indicates that a closer fit to the diagonal dotted line represents a better prediction

**Fig. 4:**
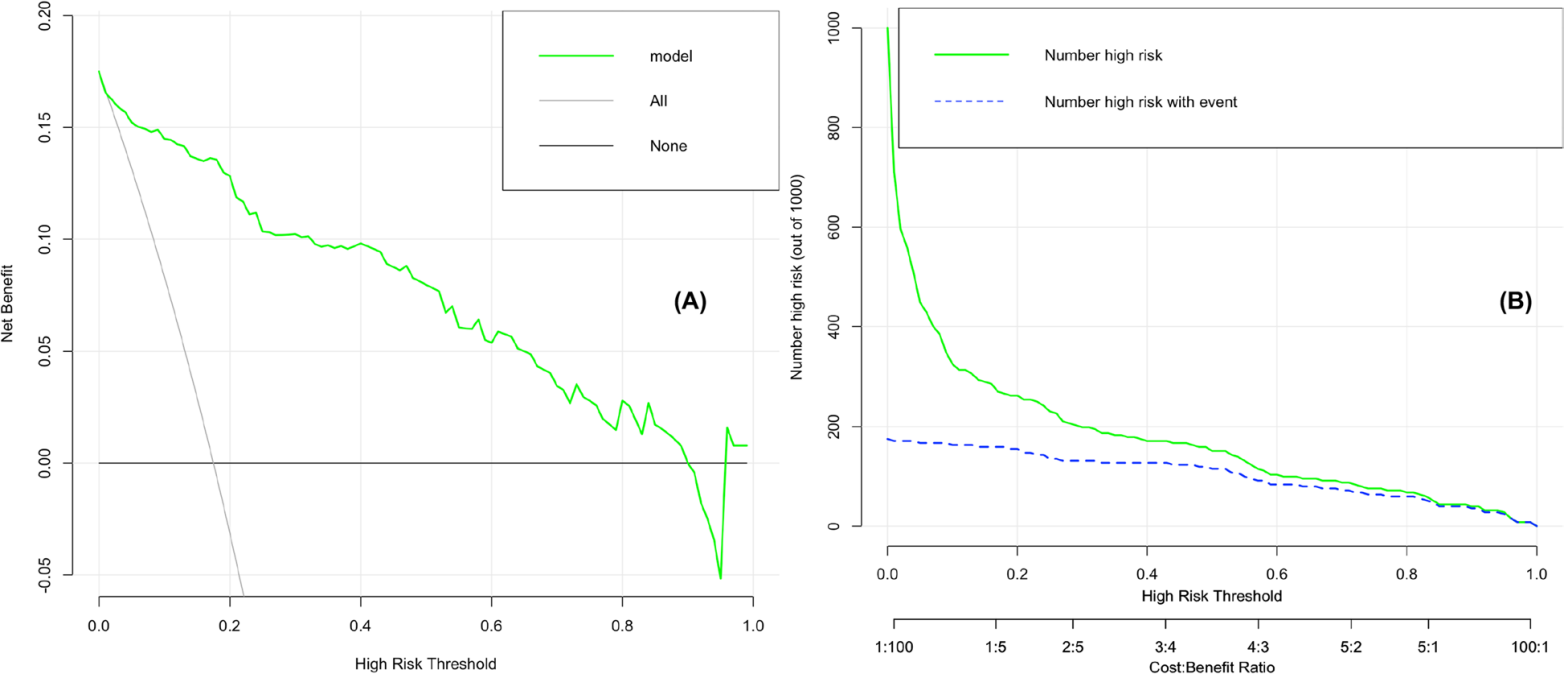
Decision curve analysis of the VA injury risk nomogram for model (**A**). The Y-axis measures the net benefit, and the X-axis is the threshold probability of requiring intervention. The thick solid line represents the assumed benefit rate of zero for all patients without VA impairment, and the thin solid line represents the magnitude of the assumed benefit rate for all patients with VA impairment. The figure shows that the risk threshold probability of visual impairment is in the range (Model 1: 3–91%), and the nomogram can be used clinically in this range. Clinical impact curves of the VA injury risk nomogram for model (**B**). The y-axis measures the number of high risks, the x-axis is the high-risk threshold, the green line for model refers to the number of people classified as positive (high risk) by the model at each threshold probability, and the blue line refers to the number of true positives at each threshold probability

The accuracy of the model in determining the presence of visual impairment was 91.2% at the optimal cut-off value (*P* = 0.187, P denotes the model-derived probability), with a sensitivity of 93.2% and a specificity of 86.5%. The Youden index was maximized, and DMI, Sub VD, DRIL, and Outer Layer Disruption were statistically significant (*P* < 0.05) factors in the model. Thus, these independent risk factors were included in the nomogram (Fig. 1). The ROC indicated that the nomogram had high discrimination (AUC = 0.931 (0.889–0.973), *P* < 0.05) (Fig. 2). Calibration curves (C-index = 0.930, *P* < 0.05) (Fig. 3) revealed good model fit consistency, and DCA illustrated that if the visual impairment risk threshold probabilities ranged from 3 to 91%, patients would significantly benefit from using this study’s model to aid decision-making. Finally, the CIC displayed the proportional number of people at risk of visual impairment at each threshold probability (Fig. 4).

## Discussion

Nomograms are considered reliable and practical predictive tools, capable of generating individual probabilities of clinical events by integrating multiple prognostic influences [15], and quantifying risk [26], thereby satisfying our desire for integrated biological and clinical models, enabling the need for personalized medicine, aiding better clinical decision-making through a user-friendly digital interface and more generalized conclusions [27]. This study concluded that DRIL and outer layer disruption (provided by OCT images of the macula), DMI and SubVD (provided by 3*3-mm blood flow images) were strongly correlated with visual impairment in patients with DR. The AUC showed that the nomogram shows better discrimination than the use of individual risk factors (Fig. 2). The visualization of influencing factors using the nomogram reveals the magnitude of influence for each risk factor for the development of visual impairment, thereby facilitating clinical discrimination of indicators that require more attention. The DCA showed that patients with risk threshold probabilities in the range of 3–91% had a higher net benefit than those in the intervention for the all patients’ scenario or the ‘no intervention at all’ scenario [15]. These findings showed that the model had a wide range of clinical applications. The CIC showed the number of true positives at risk of visual impairment at different threshold probabilities and the number predicted by the model, providing a reference for clinicians to determine the condition.

As an example of our findings, an affected eye with DRIL = 1 (21 points), DMI = 3 (31 points), Outer Layer Disruption =1 (15 points), subVD = 50% (20 points) would have a total score of 87 points, corresponding to a risk probability of substandard VA (LogMAR VA ≥ 0.5) of approximately 90% (Fig. 1). Even if patients currently had a fair vision on examination, there was a high risk that their vision would decline later; assuming a threshold probability of 20%, the DCA showed a net benefit rate of 13% in the vertical coordinate at this point, indicating that the model screened 13 additional high-risk patients for noncompliance per 100 DR patients tested for vision ((net benefit of the model - net benefit of all treatments)/(threshold probability/(1-threshold probability)) × 100), without increasing the number of false-positives [28]. CIC showed that the true number of DR patients with substandard vision was approximately 170 (out of 1000 patients) compared to the model’s predicted number of at-risk patients (260), thus indicating that the clear results are useful in clinical practice (Fig. 4). Based on the model, clinicians can implement early medical interventions, such as changing treatment regimens or increasing the frequency of follow-up visits, to reduce the risk of vision loss in DR patients, which is important for disease management in high-risk DR patients. This model can also be used for other clinical applications, such as among DR patients who need to be treated with cataract or vitrectomy surgery. If OCT/OCTA images of the macula are available, using this study’s prediction model, a general judgement can be made about the patient’s postoperative VA risk profile, which will assist in surgical planning and preoperative conversations.

The DRIL in this study was heavily represented in the model and may represent a disruption of the visual conduction pathway in the inner retina that greatly affects visual acuity in DR patients [10]. Its baseline length and degree of change over time have been shown to be correlated with VA [29]. However, the severity of DRIL has not yet been graded [30]. The relationship with visual acuity needs to continue to be explored, and the DRIL grading in the model appeared to be grade 1. The higher score than grade 2 in our study may be related to the fact that fewer eyes (1.59%) were grade 2, for reasons that will have to be explained by further research. Second, DaCosta indicated that visual acuity in DR patients gradually decreased with increasing severity of DMI [31] and that the incidence of DMI increased with increasing severity of DR [11], which was consistent with our study (Online Additional file 7). However, research on DMI is still in its infancy, and there is no definition of DMI characteristics with or without visual threat [11]. Outer layer disruption, which can be used to assess retinal photoreceptor function, was closely associated with VA [14] and was shown in this study to be an important risk factor for VA in patients with DR. Interestingly, a study suggested that microvascular changes in DR are associated with early VA loss [32],and our research also showed SubVD (provided by 3*3-mm blood flow images) were strongly correlated with visual impairment in patients with DR.

DR is still one of the leading causes of visual impairment, and the development of irreversible damage to vision should not be the ideal endpoint for all predictors but should be preceded by identification of the disease and timely intervention. The relationship between retinal changes and VA in DR patients needs to be further explored, and indicators that can more sensitively reflect visual impairment in DR patients are important for early screening and prognosis of the disease. In this study, we used multivariate stepwise regression to construct a pooled nomogram model for predicting the risk of visual impairment in DR patients based on reported risk factors for VA and new retinal structural parameters. To broaden the application of the model, we validated the feasibility and superiority of this integrated risk factor approach using VA impairment grading criteria and a multifaceted validation model using ROC, calibration plots, DCA and CIC, and the results showed that both models performed well. The entire construction and validation process was rigorous and complete, thereby providing an accurate and intuitive nomogram model for predicting the risk of visual impairment in patients with DR. This nomogram can be used to guide clinical practice.

There were also some limitations to this study. First, this was a retrospective study, and the sample size was limited. Second, DRIL was graded as occurring on one side of the central recess and both sides, and the actual length was not measured, which may also explain why the grade 2 score in the model was less than the grade 1 score. Third, this study only used data from a single visit and did not follow up on changes in patient VA; thus, our model can only predict the current risk of visual impairment and not changes in VA at a future time. Fourth, the model was not validated using an external case group. Therefore, prospective studies with larger samples and external validation are necessary to improve the model in future studies.

## Conclusion

In summary, the nomogram prediction model has demonstrated greater accuracy and utility in integrating risk factors related to VA in patients with DR. The DMI, DRIL, outer layer disruption, and SubVD are independent risk factors in the nomogram model for predicting visual impairment risk in patients with DR.

## Supplementary Information

**Additional file 1.**

**Additional file 2.**

**Additional file 3.**

**Additional file 4.**

**Additional file 5.**

**Additional file 6.**

**Additional file 7.**

## Data Availability

The datasets used and/or analyzed during the current study are available from the corresponding author on reasonable request.

## Abbreviations

DR: diabetic retinopathy
OCTA: optical coherence tomography angiography
FFA: fundus fluorescein angiography
SS-OCT: swept-source optical coherence tomography
AUC: area under the curve
ROC: receiver operating characteristic
DCA: decision curve analysis
CIC: clinical impact curves
LogMAR VA: logarithm of the minimum angle of resolution visual acuity
DMI: diabetic macular ischemia
DME: diabetic macular edema
DRIL: disorder retinal inner layer
Outer layer disruption: retinal pigment epithelium or ellipsoid zone rupture
SCP: superficial capillary plexus
DCP: deep capillary plexus
NPA: no perfusion area
FAZ: foveal avascular zone
SubVD: vessel density of choriocapillaris layer inferior
ART: arm retinal time
DN: diabetic nephropathy
UCV: ultrasound of cervical vascular
TG: triglycerides
TCH: total cholesterol
HDL-C: high density lipoprotein cholesterol
LDL-C: low-density lipoprotein cholesterol
eGFR: estimated glomerular filtration rate

## References

[1] Bhanushali D, Anegondi N, Gadde SG, Srinivasan P, Chidambara L, Yadav NK (2016). Linking retinal microvasculature features with severity of diabetic retinopathy using optical coherence tomography angiography. Invest Ophthalmol Vis Sci.

[2] Cheung N, Mitchell P, Wong TY (2010). Diabetic retinopathy. Lancet.

[3] Kang EY, Lo FS, Wang JP, Yeh LK, Wu AL, Tseng YJ (2018). Nomogram for prediction of non-proliferative diabetic retinopathy in juvenile-onset type 1 diabetes: a cohort study in an Asian population. Sci Rep.

[4] Liu Y, Yang J, Tao L, Lv H, Jiang X, Zhang M (2017). Risk factors of diabetic retinopathy and sight-threatening diabetic retinopathy: a cross-sectional study of 13 473 patients with type 2 diabetes mellitus in mainland China. BMJ Open.

[5] Mo R, Shi R, Hu Y, Hu F (2020). Nomogram-based prediction of the risk of diabetic retinopathy: a retrospective study. J Diabetes Res.

[6] Babenko B, Mitani A, Traynis I, Kitade N, Singh P, Maa AY, et al. Detection of signs of disease in external photographs of the eyes via deep learning. Nat Biomed Eng. 2022:1–14. 10.1038/s41551-022-00867-5. 10.1038/s41551-022-00867-5 PMC8963675 35352000

[7] The effect of intensive diabetes treatment on the progression of diabetic retinopathy in insulin-dependent diabetes mellitus. The Diabetes Control and Complications Trial. Arch Ophthalmol. 1995;113(1):36–51. 10.1001/archopht.1995.01100010038019. 10.1001/archopht.1995.01100010038019 7826293

[8] Youngquist RC, Carr S, Davies DE (1987). Optical coherence-domain reflectometry: a new optical evaluation technique. Opt Lett.

[9] Schwartz DM, Fingler J, Kim DY, Zawadzki RJ, Morse LS, Park SS (2014). Phase-variance optical coherence tomography: a technique for noninvasive angiography. Ophthalmology.

[10] Sun JK, Radwan SH, Soliman AZ, Lammer J, Lin MM, Prager SG (2015). Neural retinal disorganization as a robust marker of visual acuity in current and resolved diabetic macular edema. Diabetes.

[11] Cheung CMG, Fawzi A, Teo KY, Fukuyama H, Sen S, Tsai WS, Sivaprasad S. Diabetic macular ischaemia- a new therapeutic target? Prog Retin Eye Res. 2022;89:101033. 10.1016/j.preteyeres.2021.101033. Epub 2021 Dec 11. 10.1016/j.preteyeres.2021.101033 PMC11268431 34902545

[12] Campochiaro PA, Wykoff CC, Shapiro H, Rubio RG, Ehrlich JS (2014). Neutralization of vascular endothelial growth factor slows progression of retinal nonperfusion in patients with diabetic macular edema. Ophthalmology.

[13] Das R, Spence G, Hogg RE, Stevenson M, Chakravarthy U (2018). Disorganization of inner retina and outer retinal morphology in diabetic macular edema. JAMA Ophthalmol.

[14] Balaratnasingam C, Inoue M, Ahn S, McCann J, Dhrami-Gavazi E, Yannuzzi LA (2016). Visual acuity is correlated with the area of the Foveal avascular zone in diabetic retinopathy and retinal vein occlusion. Ophthalmology.

[15] Balachandran VP, Gonen M, Smith JJ, DeMatteo RP (2015). Nomograms in oncology: more than meets the eye. Lancet Oncol.

[16] Wu L, Fernandez-Loaiza P, Sauma J, Hernandez-Bogantes E, Masis M (2013). Classification of diabetic retinopathy and diabetic macular edema. World J Diabetes.

[17] Stanga PE, Tsamis E, Papayannis A, Stringa F, Cole T, Jalil A (2016). Swept-source optical coherence tomography Angio™ (Topcon Corp, Japan): technology review. Dev Ophthalmol.

[18] Soares M, Neves C, Marques IP, Pires I, Schwartz C, Costa M (2017). Comparison of diabetic retinopathy classification using fluorescein angiography and optical coherence tomography angiography. Br J Ophthalmol.

[19] La Mantia A, Kurt RA, Mejor S, Egan CA, Tufail A, Keane PA (2019). Comparing fundus fluorescein angiography and swept-source optical coherence tomography angiography in the evaluation of diabetic macular perfusion. Retina.

[20] Migicovsky Z, Harris ZN, Klein LL, Li M, McDermaid A, Chitwood DH (2019). Rootstock effects on scion phenotypes in a 'Chambourcin' experimental vineyard. Hortic Res.

[21] Sonoda S, Sakamoto T, Yamashita T, Shirasawa M, Uchino E, Terasaki H (2014). Choroidal structure in normal eyes and after photodynamic therapy determined by binarization of optical coherence tomographic images. Invest Ophthalmol Vis Sci.

[22] ETDRS Research Group Investigators. Classification of diabetic retinopathy from fluorescein angiograms. ETDRS report number 11. Early treatment diabetic retinopathy study research group. Ophthalmology. 1991;98(5 Suppl):807–22. 2062514

[23] Pascolini D, Mariotti SP (2012). Global estimates of visual impairment: 2010. Br J Ophthalmol.

[24] Fitzgerald M, Saville BR, Lewis RJ (2015). Decision curve analysis. Jama.

[25] Chen M, Li Z, Yan Z, Ge S, Zhang Y, Yang H (2022). Predicting neurological deterioration after moderate traumatic brain injury: development and validation of a prediction model based on data collected on admission. J Neurotrauma.

[26] Liang G, Chen X, Zha X, Zhang F (2017). A Nomogram to improve predictability of small-incision Lenticule extraction surgery. Med Sci Monit.

[27] Gold JS, Gönen M, Gutiérrez A, Broto JM, García-del-Muro X, Smyrk TC (2009). Development and validation of a prognostic nomogram for recurrence-free survival after complete surgical resection of localised primary gastrointestinal stromal tumour: a retrospective analysis. Lancet Oncol.

[28] Vickers AJ, Elkin EB (2006). Decision curve analysis: a novel method for evaluating prediction models. Med Decis Mak.

[29] Radwan SH, Soliman AZ, Tokarev J, Zhang L, van Kuijk FJ, Koozekanani DD (2015). Association of Disorganization of retinal inner layers with vision after resolution of center-involved diabetic macular edema. JAMA Ophthalmol.

[30] Sun JK, Lin MM, Lammer J, Prager S, Sarangi R, Silva PS (2014). Disorganization of the retinal inner layers as a predictor of visual acuity in eyes with center-involved diabetic macular edema. JAMA Ophthalmol.

[31] DaCosta J, Bhatia D, Talks J (2020). The use of optical coherence tomography angiography and optical coherence tomography to predict visual acuity in diabetic retinopathy. Eye (Lond).

[32] Sun Z, Tang F, Wong R, Lok J, Szeto SKH, Chan JCK (2019). OCT angiography metrics predict progression of diabetic retinopathy and development of diabetic macular edema: a prospective study. Ophthalmology.

