# Supplementary figures and images for: Nomogram model predicts the risk of visual impairment in diabetic retinopathy: a retrospective study
Source: BMC Ophthalmol. 2022 Dec 8;22:478. doi: 10.1186/s12886-022-02710-6 (PMC9733396; doi:10.1186/s12886-022-02710-6)

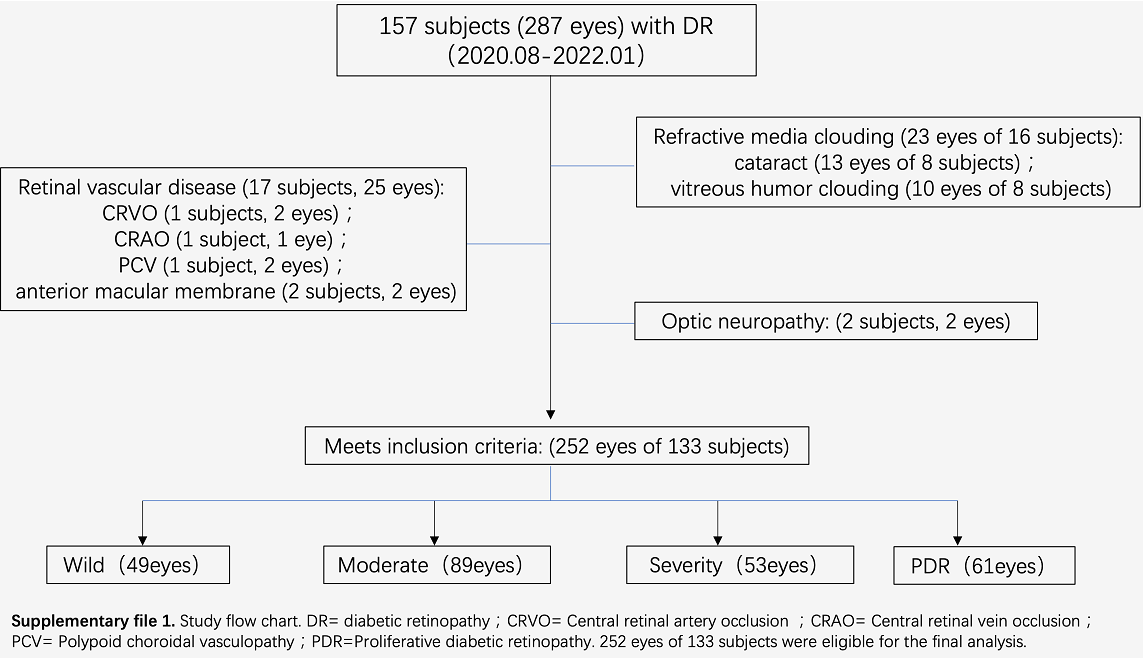

Supplement: Supplementary file 1 — Additional file 1. [file 12886_2022_2710_MOESM1_ESM.png]

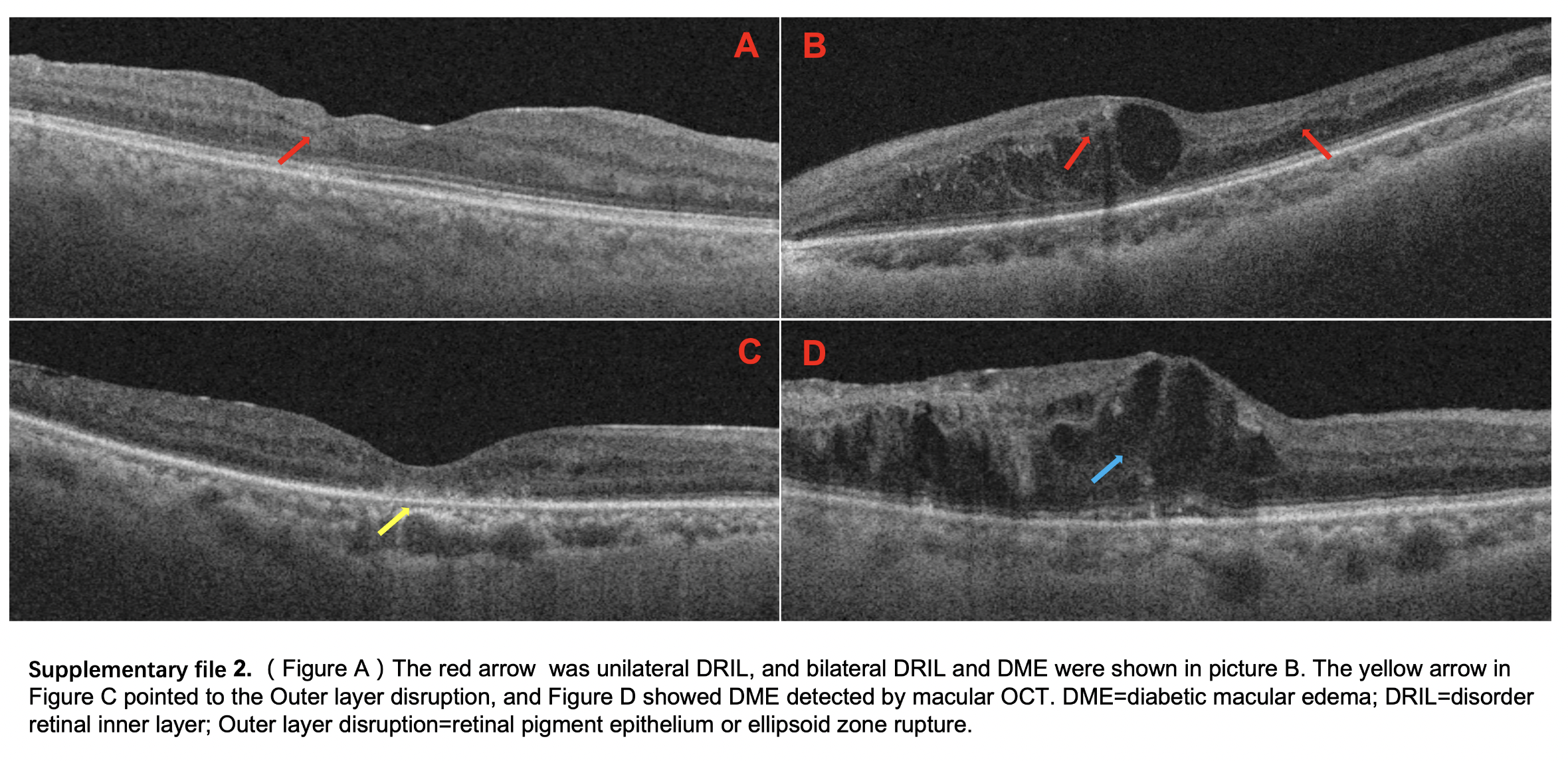

Supplement: Supplementary file 2 — Additional file 2. [file 12886_2022_2710_MOESM2_ESM.png]

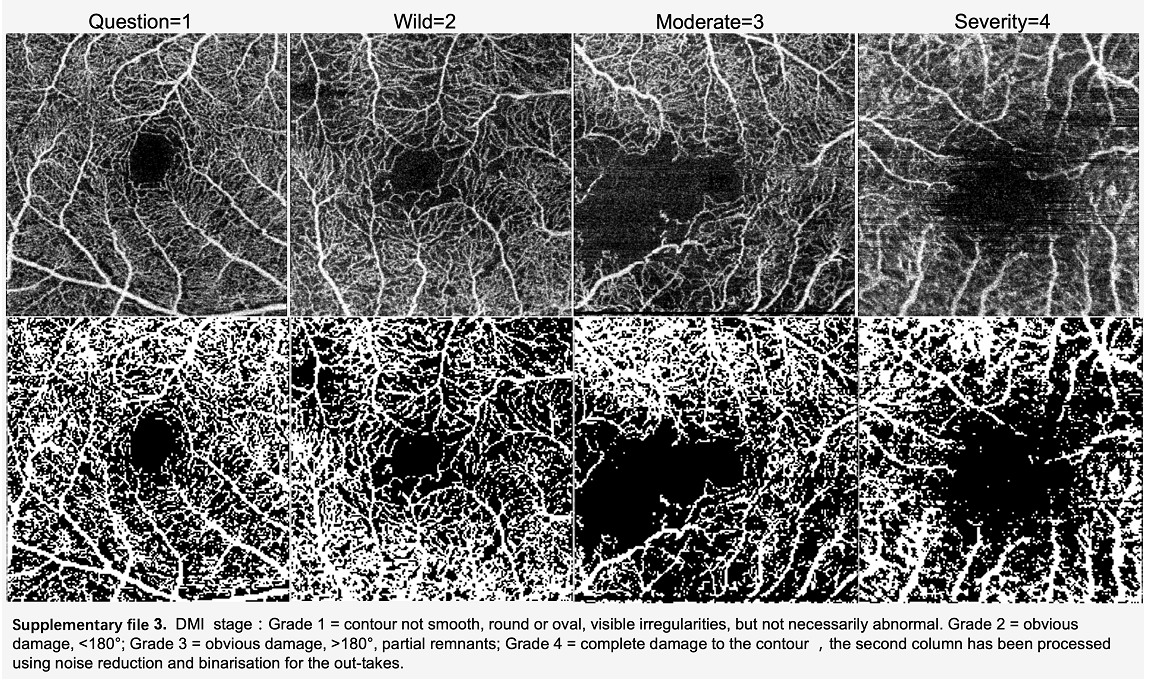

Supplement: Supplementary file 3 — Additional file 3. [file 12886_2022_2710_MOESM3_ESM.png]

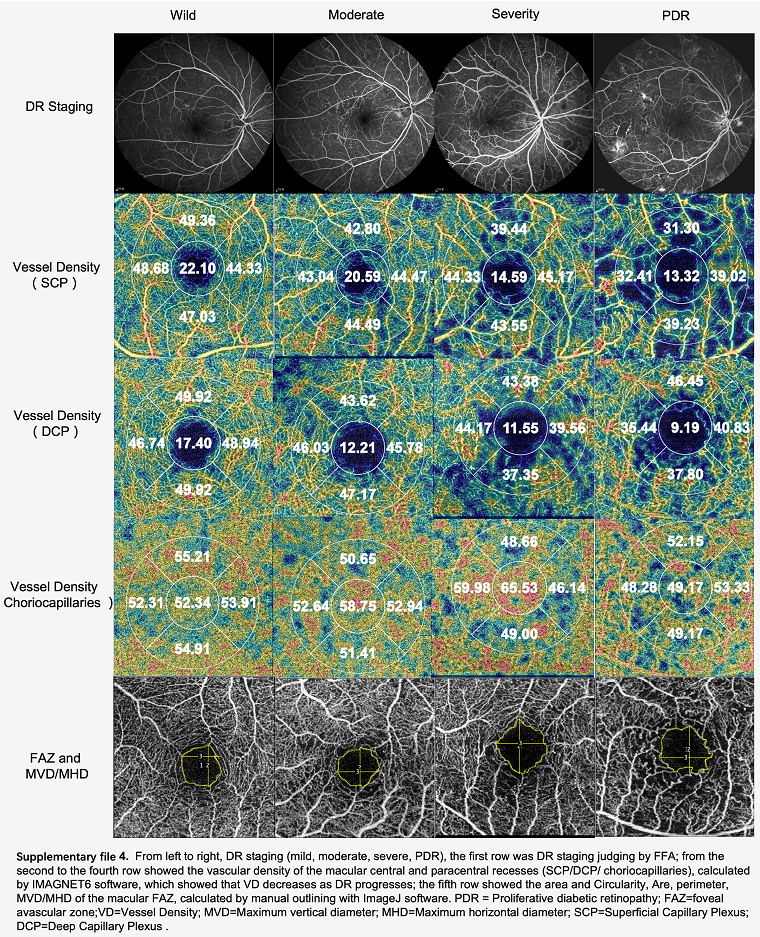

Supplement: Supplementary file 4 — Additional file 4. [file 12886_2022_2710_MOESM4_ESM.png]

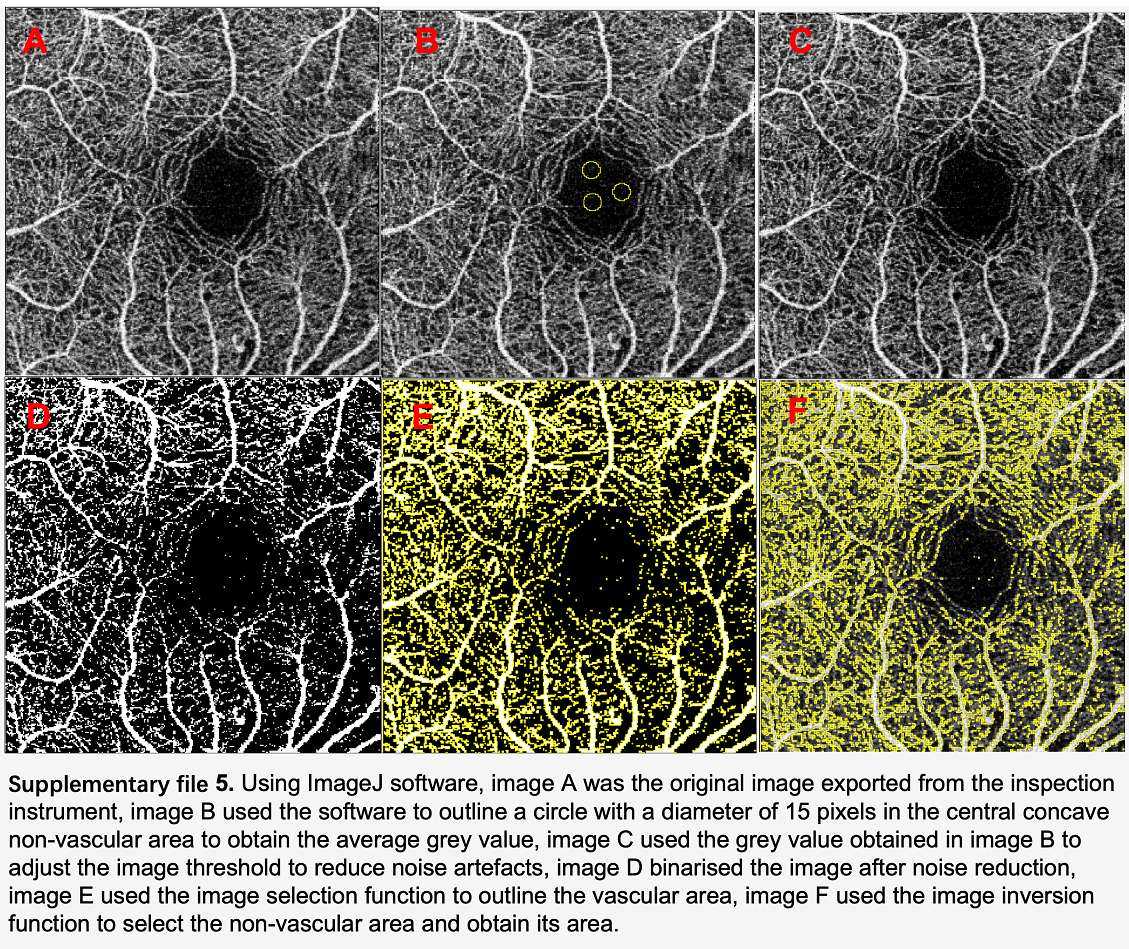

Supplement: Supplementary file 5 — Additional file 5. [file 12886_2022_2710_MOESM5_ESM.png]

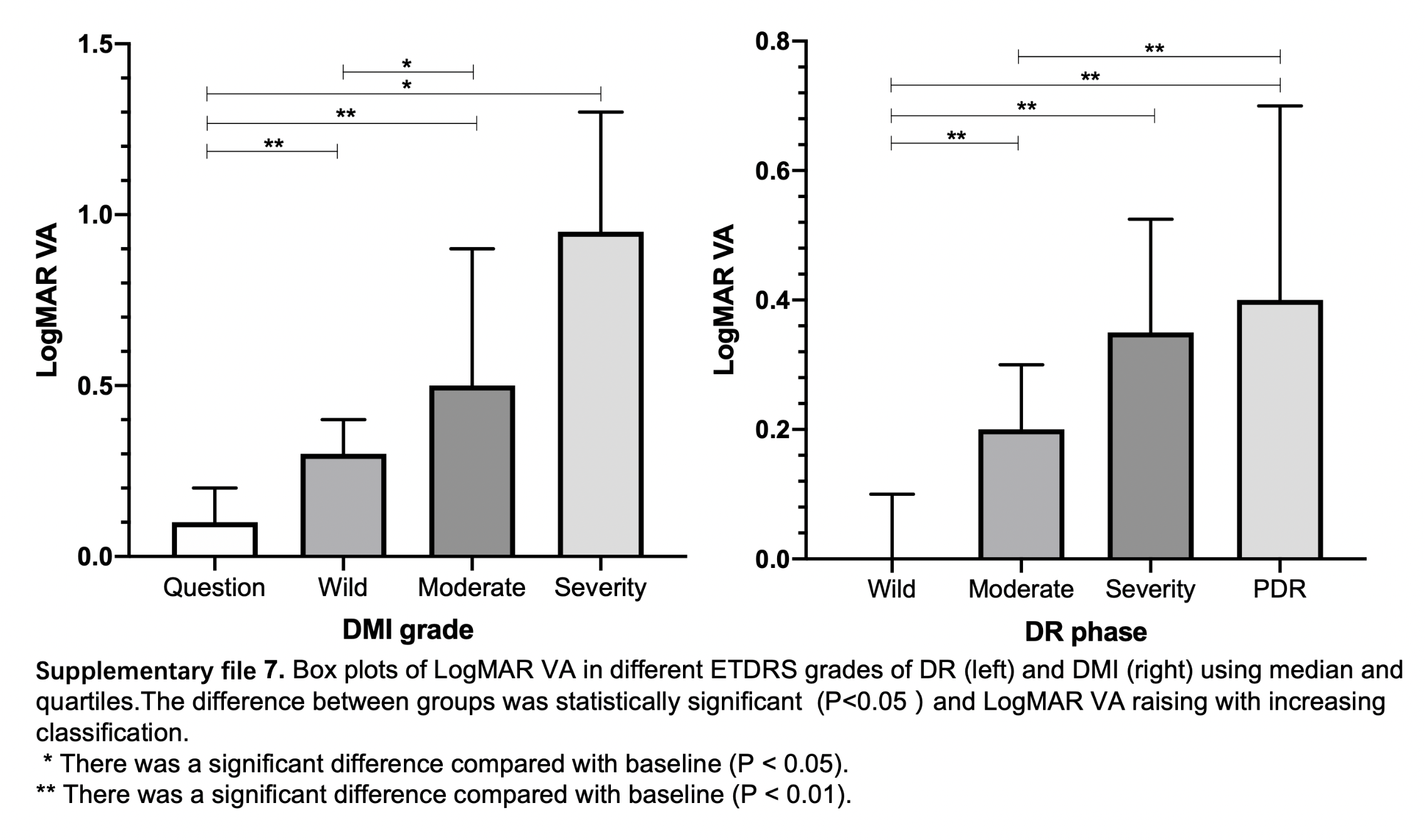

Supplement: Supplementary file 7 — Additional file 7. [file 12886_2022_2710_MOESM7_ESM.png]
