# Supplementary material for: Nomogram model predicts the risk of visual impairment in diabetic retinopathy: a retrospective study
Source: BMC Ophthalmol. 2022 Dec 8;22:478. doi: 10.1186/s12886-022-02710-6 (PMC9733396; doi:10.1186/s12886-022-02710-6)
Supplement: Supplementary file 6 — Additional file 6. [file 12886_2022_2710_MOESM6_ESM.docx]

**Supplementary file 6**. Results of consistency checks of manually measured data

| **Parameters** |  | ICC | 95% confidence interval | | P |
| --- | --- | --- | --- | --- | --- |
| **Foveal Avascular Zone** |  |  |  |  |  |
| Area of SCP |  | 0.996 | 0.995 | 0.997 | 0.000 |
| DCP |  | 0.985 | 0.981 | 0.988 | 0.000 |
| Perimeter of SCP |  | 0.984 | 0.979 | 0.987 | 0.000 |
| DCP |  | 0.965 | 0.955 | 0.973 | 0.000 |
| Circularity of SCP |  | 0.940 | 0.923 | 0.953 | 0.000 |
| DCP |  | 0.206 | 0.085 | 0.321 | 0.000 |
| MVD of SCP |  | 0.972 | 0.965 | 0.978 | 0.000 |
| DCP |  | 0.831 | 0.788 | 0.866 | 0.000 |
| MHD of SCP |  | 0.973 | 0.965 | 0.979 | 0.000 |
| DCP |  | 0.886 | 0.856 | 0.910 | 0.000 |
| **Non-perfusion Area** |  |  |  |  |  |
| 3*3mm of SCP |  | 0.999 | 0.999 | 0.999 | 0.000 |
| DCP |  | 0.994 | 0.992 | 0.995 | 0.000 |

MVD=Maximum vertical diameter; MHD= maximum horizontal diameter; NPA=Non-perfusion Area; FAZ=Foveal Avascular Zone; ICC =intraclass correlation efficient.
